# Analysis of the Feasibility of a Vaccination Campaign against Influenza Epidemic and COVID-19 Pandemic in French Emergency Departments: A National Survey

**DOI:** 10.3390/vaccines9040400

**Published:** 2021-04-19

**Authors:** Daniel Aiham Ghazali, Christophe Choquet, Donia Bouzid, Luisa Colosi, Arsalene Ben Hammouda, Mathias Wargon, Matthieu Gay, Prabakar Vaittinada Ayar, Bendecite Douay, Eric Revue, Louis Soulat, Romain Hellmann, Enrique Casalino

**Affiliations:** 1 Emergency Department and EMS, Assistance Publique-Hôpitaux de Paris (AP-HP), University Hospital of Bichat, 75018 Paris, France; Christophe.choquet@aphp.fr (C.C.); donia.bouzid@aphp.fr (D.B.); luisa.colosi@aphp.fr (L.C.); Romain.HELLMANN@ars.sante.fr (R.H.); enrique.casalino@aphp.fr (E.C.); 2 The French National Study Group for Efficiency and Quality of Emergency Departments and Non-Scheduled Activities, Université de Paris, 75018 Paris, France; abenhammouda@ch-cotefleurie.fr (A.B.H.); mathias.wargon@ch-stdenis.fr (M.W.); eric.revue@aphp.fr (E.R.); 3 IAME (Infection, Antimicrobial, Modeling, Evaluation), INSERM UMR1137, Université de Paris, 75018 Paris, France; 4 Emergency Medical Services, Hôpital of Beaujon, 92110 Clichy, France; benedicte.douay@aphp.fr; 5 Emergency Department, Hôpital de la Côte Fleurie, 14113 Cricquebœuf, France; 6 Emergency Department, Hôpital Delafontaine, 93200 Saint Denis, France; 7 Emergency Department, Hôpital Beaujon, 92110 Clichy, France; matthieu.gay@aphp.fr (M.G.); prabakar.vaittinadaayar@aphp.fr (P.V.A.); 8 Emergency Department, Assistance Publique-Hôpitaux de Paris (AP-HP), Hôpital Lariboisière, 75010 Paris, France; 9 Emergengy Departement, SAMUSMUR Urgences, CHU Rennes, Hôpital PONCHAILLOU 35 000 RENNES, France; Louis.SOULAT@chu-rennes.fr; 10 Regional Health Agency of Ile de France, 93200 Saint Denis, France

**Keywords:** influenza, COVID-19, vaccination campaign, emergency departments, healthcare workers, patients

## Abstract

Background: Vaccination is one of the most effective ways to fight the influenza epidemic and the coronavirus disease 2019 (COVID-19) pandemic, which represent a major public issue. The objective was to investigate the adherence of heads of French emergency departments (ED) and nursing departments on a potential vaccination campaign of healthcare workers (HCW) and patients in ED. Method: In February 2021, ED and nursing department heads were asked to answer a national survey. It included 24 questions designed to cover some dimensions, including characteristics of the hospital and emergency departments (ED) and questions on vaccination. Results: 414 responses out of 800 questionnaires (51.8%) were collected. Scores out of 10 were, respectively, 7 (6–8) and 8 (6–9) for vaccination against influenza and COVID-19 for HCW and 2 (2–3) and 2 (2–4) for ED patients (H = 989.3; *p* < 0.0001). Multivariate logistic regression found that the existence of a vaccine program in the hospital and the use of point of care influenza PCR in ED were positively associated with the acceptance of influenza vaccination campaign for HCW (*p* = 0.003) and patients (*p* = 0.015). Factors limiting adherence to a vaccination program of HCW and patients were lack of medical staff (*p* = 0.041 for HCW and *p* < 0.0001 for patients), overcrowded ED (*p* < 0.001), and the inability to follow up with patients after the ED visit (*p* < 0.0001). Conclusions: There have been many missed opportunities for influenza vaccination, and there is pressure to vaccinate against COVID-19 as soon as possible. Vaccination campaigns in ED could help to improve vaccination coverage. ED staff are more likely to vaccinate HCW than patients. There are factors that support the implementation of such programs, which can be grouped into a culture of diagnosis, control, and prevention of viral infectious diseases within the hospital and ED. On the other hand, there are limiting factors, such as overcrowding and lack of personnel.

## 1. Introduction

**Figure A1:**
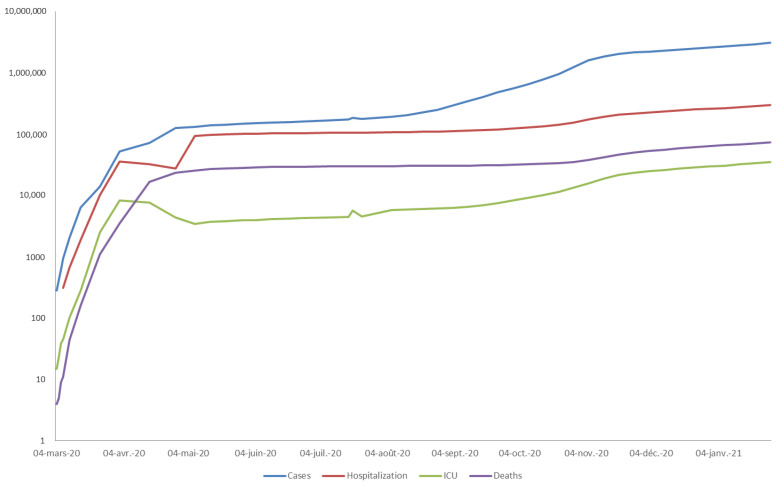
Evolution of the COVID-19 pandemic waves in France (March 2020—January 2021).

The seasonal influenza epidemic remains a major public health issue with 3–5 million severe cases resulting in up to 650,000 deaths annually [1]. Every winter, the seasonal flu affects 2 to 8 million people in France, causing several thousand deaths, mainly elderly people or patients with chronic diseases [2]. On the other hand, 2020–2021 are exceptional years in terms of global health crisis due to the coronavirus disease 2019 (COVID-19) pandemic. SARS-CoV-2, the virus, is responsible for COVID-19, which was first reported in China and then became a worldwide pandemic [3]. The COVID-19 pandemic is an emergency of international concern [4]. France has been confronted with two pandemic waves, one in spring 2020 and the other since autumn 2020 (Figure A1). France has experienced significant regional disparities, from 3.6% to more than 20% seroprevalence of anti-COVID-19 antibodies. The Grand Est region and the Ile de France region were the most affected in France by the COVID-19 pandemic [5]. Considering its high mortality and rapid spread, effective action was urgently needed [6]. The control, not only of influenza epidemics, but also of COVID-19, depends on preventive barrier and hygiene measures. Nevertheless, vaccination is certainly the most effective way to fight against these viruses [7]. Influenza vaccination coverage rates in populations at risk for severe disease vary widely around the world, ranging from 10% to 80% [8,9]; low rates are partially due to missed opportunities to vaccinate [10]. With regard to vaccination against COVID-19, the race is on to vaccinate the population. Vaccination began the last week of December 2020 and, as of March 5, has been given to about 4,000,000 people [11]. The French government is expanding vaccination training to various trained health workers to accelerate the pace of vaccination. On the other hand, according to the Ministry of Health, in 2019 there were 19,714,060 emergency department (ED) visits [12]. In this context, the aim of this study was to investigate the perception of EDs regarding the possibility of vaccinating patients with the seasonal influenza and COVID-19 vaccinations. The study hypothesis is that there may be a difference in acceptability because influenza vaccination is an annual public health mission, whereas the world is currently experiencing an unprecedented health crisis due to the COVID-19 pandemic.

## 2. Methods

### 2.1. Study Design

In this cross-sectional study that was undertaken in all EDs in France, ED and nursing department heads were requested to answer an electronic survey. In France, 636 hospitals have Eds, including 473 public hospitals, 36 private non-profit hospitals, and 127 private for-profit hospitals. Of these hospitals, 59% EDs receive less than 30,000 patients per year. Adult EDs number 547, while there are 73 mixed adult and pediatric EDs and 16 pediatric EDs [13]. On 12 February 2021, an electronic survey was distributed via email. Emails were compiled by using the following lists: study group for efficiency and quality of EDs and non-scheduled activities departments, as well as academic and hospital associations [13]. They were contacted on 12 February 2021. They received reminders for filling out the survey the next day and then every two days until 26 February 2021. There was an informatics check for duplication to prevent participants from responding more than once. ED department heads were asked to share the survey with nurse supervisors and physicians with responsibility for disaster response. The survey (Appendix B) included 24 questions designed to cover characteristics of the hospital and ED, management, resources, ED visits, and questions on a potential adherence to the influenza and COVID-19 vaccination campaign of healthcare workers (HCW) and patients with indication to be vaccinated. The questionnaire asked about the activity of the department, which could influence the perception of whether or not a vaccination campaign would be carried out in the ED. The questions also aimed to assess whether there was a culture of vaccination in the ED and in the hospital. Similarly, the participants were asked about the current practice of PCR testing in their ED (tetanus, influenza, and COVID-19), based on the assumption that such a practice could modify adherence to the implementation of a vaccination campaign in the ED. These questions were validated by a committee of experts using the Delphi method, wherein questions were added, removed, or modified until a consensus of at least 65% agreement was reached [13]. The experts were ED department heads with a university degree in management teaching at the University of Paris. After validation, the survey was sent to the participants.

### 2.2. Data Analysis

The Shapiro–Wilk test was used to assess data distribution. Continuous variables are presented as mean ± SD and, if necessary, as median and interquartile range (Q1–Q3); and categorical variables, such as number and percentage. Comparative analysis between several questions used ANOVA or Kruskal–Wallis non-parametric test to test the equality of multiple scores. In case of statistically significant results, Scheffe post hoc test was pre-specified to explore differences between multiple mean scores while controlling the experiment-wise error rate. Chi-square test, or McNemar Chi-square test, was used to compare categorical data. Univariate and multivariate logistic regression were used to determine factors associated with the willingness of ED medical and paramedical staff to vaccinate HCW and patients against influenza and COVID-19. Then, multiple logistic regression was conducted to determine the factors that might predispose respondents to agree to vaccinate HCW and patients. Factors, for which a median score of 0 to 10 was greater than the median score, related to the agreement to vaccinate HCW and patients were retained to perform the multivariate regression. Variables with *p* < 0.2 in univariate analysis were included in the multivariate stepwise logistic regression model to determine those related to influenza and COVID-19 vaccination.

All in all, factors that were hypothesized to impact ED caregivers to vaccinate hospital health workers and/or patients against influenza and/or COVID-19 were: geographic location of the hospital, professional status of the caregiver (medical or nursing), type of ED (adult, pediatric, or mixed), number of ED visits, characteristics of ED, existence of a vaccination center, whether or not the health workers were vaccinated against influenza in the hospital, whether or not patients were vaccinated against influenza in the hospital, existence or not of an infectious disease department in the hospital, whether or not the ED vaccinated HCW and/or patients against influenza, use of influenza PCR, use of point-of-care influenza PCR in the ED, use of influenza and/or tetanus antigen tests in the ED, and performance of tetanus vaccination in the ED. The other factors analyzed were the lack of follow-up of ED patients, the ED’s lack of medical staff, the team’s adherence to a possible vaccination campaign, the refusal of certain patients to be vaccinated, and lack of vaccines available on the site. *p* value of <0.05 was considered significant. All statistical analysis was conducted using Statistica v12 software.

### 2.3. Ethics Statement

Data collection and storage were approved by the French National Commission for Data Protection and Liberties. All data were completely anonymous, and the study was conducted in accordance with the 1964 Helsinki Declaration. The Emergency Ethics Committee for Biomedical Research of Assistance Publique-Hôpitaux de Paris approved this study (study number: DAG-3-BCH-21). No ethical number was required. Participants were informed about the objectives and the method. Answering the questionnaire was considered as agreeing to the terms of the study.

## 3. Results

In all, 414 responses out of 800 questionnaires (51.8%) were collected. All the responses were included in the analysis. Since all questions were mandatory, there were no missed responses. A little more than half of the participants worked in Paris (21.50%) or the Paris region (36.23%). The other participants came from 40 other French departments, including the overseas departments. Among the respondents, there were 356 (85.58%) physicians, including 82 (23.03%) ED heads and 58 (14.42%) nursing department heads. The characteristics of EDs and hospitals where they were working are given in Table 1.

**Figure 1:**
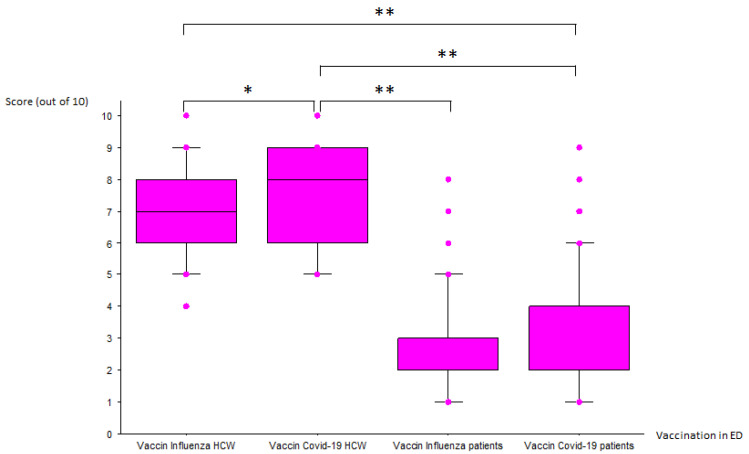
Assessment of vaccination as part of the mission of EDs according to the respondents (*n* = 414). HCW: healthcare workers; Vaccin: vaccination; * *p* < 0.01; ** *p* < 0.001.

Participants were asked to respond with a score of 0 to 10 whether they thought that influenza and COVID-19 vaccination of HCW and patients could be part of the ED’s mission. Scores are given in Figure 1. Scores out of 10 were respectively 7 (6–8) and 8 (6–9) against influenza and COVID-19 for HCW and 2 (2–3) and 2 (2–4) against influenza and COVID-19 for patients with indication to be vaccinated. These four propositions of vaccination were significantly different (H = 989.3; *p* < 0.0001). Significant differences were found between HCW and patients for the two vaccines (*p* < 0.0001) and between the two vaccines (against influenza and COVID-19) in the HCW group (*p* = 0.0003).

As presented in Table 2, several factors are associated with potential adherence or non-adherence to a vaccination campaign. The factors that seem to positively influence any form of vaccination are implementation of a previous vaccination campaign (*p* < 0.05) and use of point of care PCR in the ED (*p* < 0.05). Factors that seem to negatively influence any form of vaccination (especially for patient vaccination) were overload of clinical activity (*p* < 0.0001) and lack of staff (*p* < 0.0001). Lastly, lack of vaccine available in the hospital was negatively associated with the development of a vaccination strategy (*p* < 0.0001).

In multivariate analysis (Table 3), it appeared that emergency teams with point of care influenza PCR were more likely to be in favor of conducting an influenza vaccination campaign for HCW [OR 1.85 (1.23–2.79), *p* = 0.003] and patients [OR 1.77 (1.11–2.80), *p* = 0.015]. Professionals practicing in Paris hospitals were more likely to agree to vaccinate HCW against COVID-19 than in other regions of France [OR 0.46 (0.30–0.71), *p* = 0.0004]. Nursing department heads were significantly more willing than medical teams to vaccinate HCW against influenza [OR 0.54 (0.30–0.99), *p* = 0.046] and also to vaccinate patients against influenza [OR 0.33 (0.18–0.62), *p* = 0.0006] and COVID-19 [OR 0.24 (0.12–0.47), *p* < 0.0001]. Lack of medical staff was a factor limiting adherence to vaccinating HCW against COVID-19 [OR 0.61 (0.38–0.98), *p* = 0.041] and patients against influenza [OR 0.33 (0.20–0.53), *p* < 0.0001] and COVID-19 [OR 0.27 (0.15–0.47), *p* < 0.0001]. ED overcrowding was a limiting factor for possible vaccination against the annual influenza epidemic of HCW [OR 0.34 (0.17–0.68), *p* = 0.002] and patients [OR 0.26 (0.12–0.56), *p* = 0.0006]. Lastly, the inability to follow up with patients after the ED visit was an extremely limiting factor for adherence to an influenza [OR 0.34 (0.20–0.57), *p* < 0.0001] and COVID-19 vaccination program for patients [OR 0.21 (0.12–0.37), *p* < 0.0001]. Other factors are given in Table 3.

## 4. Discussion

The present study aimed to explore the feasibility of vaccinating HCW, and patients in EDs with indication to be vaccinated against influenza and COVID-19. We were interested in the influenza virus, which is recurrent every year and responsible for annual deaths. This mission could be considered a public health mission, especially since there have been many missed opportunities to vaccinate patients [2]. For HCW, some studies even suggest that vaccination be mandatory so as to increase coverage [14]. Moreover, simplifying access to vaccination through decentralizing vaccination centers and providing incentives for HCW can improve the vaccination rate [15]. The vision may be different for the COVID-19 virus, which is responsible for an exceptional pandemic that is causing an unprecedented global health crisis. Seasonal influenza epidemics are responsible for a high number of severe forms of flu and deaths in unvaccinated patients with indication to be vaccinated, especially in cases where the indications for the vaccine are recognized [16], impacting the health care system with additional costs [17]. In a previous study in a Parisian academic hospital, it was found that 24% of the patients admitted in the ED during the early- and epidemic seasonal influenza period were at high risk for severe influenza, while only one third of them were vaccinated against influenza. Furthermore, missed opportunities for vaccination concerned nearly 70% of emergency patients [2]. On the other hand, 2020 and 2021 are exceptional years with an international COVID-19 pandemic that globally, by 12 March 2021, affected 118,058,503 patients, including 2,621,046 deaths reported to WHO [18]. As of 10 March 2021, a total of 300,002,228 vaccine doses have been administered. France is currently facing its second wave of the COVID-19 pandemic, which is responsible for 3,894,447 cases including 89,077 deaths according to the WHO [18]. As of 9 March 2021, 4,164,418 people have received at least one dose of COVID-19 vaccine, representing 6% of the overall population [19]. However, many patients who need to be vaccinated come to the ED and could, therefore, be vaccinated during their visit, unless they are suffering from an acute pathology contraindicating the vaccination. The present study therefore aimed to assess, by means of a survey of ED managers in France, the possibility of vaccinating HCW and patients who present to the ED and who have an indication to be vaccinated against influenza and/or COVID-19. Respondents were found to be significantly more supportive of vaccinating HCW than vaccinating patients. Among HCW, they were significantly more likely to vaccinate caregivers against COVID-19 than against influenza.

Univariate and multivariate analysis explain these differences. Factors that inhibit the ability to vaccinate patients appeared to be overcrowding, lack of medical staff, and lack of patient follow-up. Lack of follow-up of vaccinated persons was not found for HCW. The lack of difficulty in following up with colleagues may explain the more favorable opinion about vaccinating HCW compared with vaccinating patients. Logically, the factors of lack of medical staff and overload of activity were found to limit the realization of any vaccination campaign. However, a recent study showed that a vaccination campaign could be carried out in the ED without impacting the clinical activity of the department. This campaign in a French ED led to an increase of influenza vaccination coverage from 32.2 to 65.9% [20], while many multi-strategy campaigns increased vaccination coverage from 4 to 10% [21,22,23,24,25]. The strategy, developed to improve vaccination coverage, was for doctors and nurses to propose, several times if necessary, immediate vaccination during the ED consultation. In addition, the vaccine was immediately available and could be quickly administered by the nurses [20]. This strategy of vaccination by nurses could be a solution for developing vaccination programs in EDs. Indeed, our study seems to find that the lack of medical staff was a hindrance, whereas this did not seem to be the case in nursing departments. In addition, nurses seemed to be more willing to participate in a vaccination campaign than physicians. It is possible that they consider there is less of a staffing problem, and that, contrary to physicians, nurses routinely inject medication. Moreover, in univariate analysis, this study demonstrated the value of having a vaccine available to promote adherence to the vaccination program. This is consistent with the findings of Casalino et al., who hypothesized a significant increase in vaccination coverage because the vaccine was immediately available [20].

The factors that seem to favor adherence to a vaccination campaign addressed to HCW and patients seemed to be linked to an institutional and ED culture of combatting infectious viral diseases. For example, when point of care influenza PCR was carried out in the ED, and when vaccination campaigns are carried out in the hospital and in the ED, the acceptability of a vaccination campaign was promoted. Surprisingly, we found that respondents from Paris and the Paris region were more likely to support a vaccination campaign. One hypothesis could be that the Paris region was one of the most affected by the COVID-19 pandemic during the first wave. However, this hypothesis is limited by the fact that more than half of the respondents were from Paris and the Paris region (Ile de France) and by the fact that there was not much response from the Grand Est region, which is the other region that was severely affected by the COVID-19 pandemic [5]. Furthermore, this study was conducted during the second wave, during which other regions were markedly affected by COVID-19. The present study should be completed by analyzing other parameters that could influence a team’s adherence to a possible vaccination campaign. Recent HCW education and experience are factors that could influence the acceptability of a vaccine for themselves [26] and, therefore, potentially influence adherence to a vaccination program of other caregivers and emergency patients. Increasing caregivers’ awareness of the need to vaccinate would increase adherence to the implementation of vaccination campaigns [27]. In the study by Pichon et al., the authors also discussed the importance of training in influencing the adoption of the vaccine. They also highlighted other factors that should be analyzed, such as vaccination status and care for at-risk patients. These factors are indeed two parameters strongly influenced by a social desirability bias that can impact beliefs and adherence to the vaccination campaign [28]. In addition, HCW already vaccinated against influenza were more in favor of vaccination and even of it becoming mandatory [28].

Finally, the choice of participants was based on the fact that, in March, doctors and nurses could possibly vaccinate in France. The questionnaire therefore targeted managers and leaders in EDs. Nevertheless, as the health crisis evolves into a third wave, vaccination skills could be extended to other categories of health personnel. It might be interesting to address the survey to these categories to assess adherence to mass vaccination campaigns.

### Limitations

The present study is not without limitations. First, this survey was voluntary and not all EDs responded. Respondents may be those most motivated to answer. The rate of unanswered questionnaires was high but hardly surprising in such surveys [28]. Slightly more than half of the responses came from the Paris region, which is, therefore, over-represented compared to the other regions. This may create a bias in the weight of the responses. However, this bias is mitigated by the fact that this is the most populated region in France; the general population of this region (12.3 million inhabitants) represents 18% of the general population. Finally, there is a lack of data on non-responding EDs, which might have had a different point of view from those who answered.

## 5. Conclusions

The seasonal influenza epidemic and COVID-19 pandemic are responsible for heavy morbidity and mortality and represent a major public health issue. Vaccination is one of the most effective ways to fight these epidemics. However, there have been many missed opportunities for influenza vaccination and there is pressure to vaccinate against COVID-19 as soon as possible. Vaccination campaigns in EDs could help improve vaccination coverage. This French national survey demonstrates that ED staff are more likely to vaccinate HCW than to vaccinate patients. There are factors that support the implementation of such programs, which can be grouped into a culture of diagnosis, control, and prevention of viral infectious diseases within the hospital and ED. On the other hand, there are limiting factors, such as overcrowding and lack of personnel. This survey highlights elements for developing future vaccination campaigns against influenza and COVID-19.

## Figures and Tables

**Table 1:** Hospitals and ED characteristics.

Characteristics	*n* (414)	%
Type of hospital		
University hospital	174	42.03
General hospital	216	52.17
Private hospital	24	5.80
Type of ED		
Adults	311	75.12
Mixed (adults and pediatrics)	83	20.05
Pediatrics	20	4.83
Number of ED visits (per year)		
More than 60,000	133	32.13
From 30,000 to 60,000	214	51.69
Less than 30,000	67	16.18
ED situation according to the respondents		
The hospital’s downstream ED is inadequate	176	42.51
Patients are often in the ED waiting for a hospital bed	79	19.08
None of the above	18	4.35
The ED’s facilities are inadequate	55	13.29
Waiting times are long	20	4.83
ED is very often overcrowded	66	15.94
Infectiology and vaccination		
The hospital has a vaccination center	340	82.13
The hospital vaccinates HCW against influenza	403	97.34
The hospital vaccinates patients against influenza	93	22.46
The hospital has an Infectious Diseases Department	236	57.00
The ED vaccinates its HCW against influenza	355	85.75
The ED vaccinates the patients against influenza	49	11.84
Influenza PCR is available for the ED	341	82.37
Point of care influenza PCR is available in the ED	193	46.62
Point of care influenza antigen test is available in the ED	146	35.27
Rapid test for tetanus antibodies is available in the ED	361	87.20
Tetanus vaccine is available in the ED	334	80.68

ED: emergency department; HCW: Healthcare workers; PCR: polymerase chain reaction.

**Table 2:** Predictors of adherence to a potential vaccination campaign against Influenza and COVID-19 in univariate analysis.

Factors	HCW against Influenza		HCW against COVID-19		Patients against Influenza		Patients against COVID-19	
	OR	*p*	OR	*p*	OR	*p*	OR	*p*
Geographic location of the hospital	0.47	<0.001	0.34	<0.001	0.76	0.2	0.77	0.21
Professional status of respondent ^1^	0.61	0.08	0.92	0.81	0.45	0.005	0.43	0.003
Type of ED ^2^	0.79	0.20	0.84	0.30	0.75	0.13	0.92	0.67
Type of hospital ^3^	0.73	0.06	0.71	0.04	0.61	0.005	0.63	0.009
Number of ED visits ^4^	0.62	0.001	0.43	<0.001	0.86	0.31	0.75	0.06
Characteristics of the ED ^5^	0.94	0.26	0.97	0.55	0.98	0.65	1.02	0.75
Existence of a vaccination center in the hospital	1.81	0.02	1.64	0.53	0.94	0.83	0.85	0.53
Vaccination of HCW against influenza in the hospital	1.19	0.70	0.95	0.94	0.69	0.55	0.98	0.98
Vaccination of patients against influenza in the hospital	0.98	0.80	1.43	0.13	2.23	<0.001	1.85	0.01
Existence of an infectiology service in the hospital	1.46	0.06	1.82	0.003	1.02	0.94	1.00	0.99
Vaccination of HCW against influenza in the ED	1.97	0.01	2.78	<0.001	2.55	0.003	1.97	0.03
Vaccination of patients against influenza in the ED	0.80	0.51	1.94	0.03	5.30	<0.001	2.44	0.004
Use of influenza PCR in the hospital	0.96	0.91	1.14	0.61	0.57	0.03	0.62	0.07
Use of point-of-care influenza PCR in the ED	1.87	0.002	1.54	0.03	2.15	<0.001	1.56	0.03
Use of influenza antigen tests in the ED	0.97	0.92	0.81	0.4	1.22	0.35	1.17	0.46
Use of tetanus antibodies tests in the ED	1.34	0.32	0.86	0.61	2.45	0.007	1.35	0.34
Performance of tetanus vaccination in the ED	0.99	0.96	0.81	0.41	1.81	0.027	0.92	0.75
Lack of follow-up of ED patients	1.22	0.01	1.20	0.12	1.88	<0.001	2.04	<0.001
Overcrowding in ED	1.37	0.01	1.32	0.009	1.99	0.03	2.70	<0.001
Lack of medical staff	1.22	0.03	1.28	0.006	1.87	<0.001	2.50	<0.001
Lack of paramedical staff	1.12	0.14	1.14	0.29	1.67	0.002	1.96	<0.001
Team’s adherence to a possible vaccination campaign	0.86	0.34	1.27	0.003	0.99	0.37	1.36	0.005
Refusal of certain patients to be vaccinated	0.98	0.26	1.24	0.99	0.92	0.05	0.94	0.29
Lack of vaccine available in the hospital	0.90	0.51	1.42	0.01	1.20	0.04	2.02	<0.001

ED: emergency department; HCW: healthcare workers; PCR: polymerase chain reaction; ^1^ Physician or nurse; ^2^ adult, pediatric, or mixed; ^3^ university hospital, general hospital or private; ^4^ <30,000/year, 30,000–60,000/year, or >60,000; ^5^ characteristic of ED in terms of premises, equipment, delays, overload of clinical activity.

**Table 3:** Predictors of adherence to a potential vaccination campaign against Influenza and COVID-19 in multiple logistic regression.

	Odds Ratio	IC95%	*p*
HCW vaccination against Influenza in ED (*n* = 414)			
Professional status of respondent ^1^	0.54	0.30–0.99	0.046
Existence of an infectiology service in the hospital	1.60	1.05–2.45	0.029
Use of point-of-care influenza PCR in the ED	1.85	1.23–2.79	0.003
Overcrowding in ED	0.34	0.17–0.68	0.002
HCW vaccination against COVID-19 in ED			
Geographic location of the hospital	0.46	0.30–0.71	<0.001
Number of ED visits ^2^	0.49	0.30–0.79	0.003
Lack of medical staff	0.61	0.38–0.98	0.041
Team’s adherence to a possible vaccination campaign	0.47	0.29–0.76	0.002
Patients’ vaccination against Influenza in ED			
Professional status of respondent ^1^	0.33	0.18–0.62	<0.001
Vaccination of patients against influenza in the hospital	2.04	1.17–3.56	0.012
Vaccination of patients against influenza in the ED	3.80	1.78–8.11	0.0005
Use of point-of-care influenza PCR in the ED	1.77	1.11–2.80	0.015
Lack of follow-up of ED patients	0.34	0.20–0.57	<0.001
Lack of medical staff	0.33	0.20–0.53	<0.001
Patient vaccination against COVID-19 in ED			
Professional status of respondent ^1^	0.24	0.12–0.47	<0.001
Vaccination of patients against influenza in the hospital	2.01	0.95–4.25	0.042
Vaccination of patients against influenza in the ED	2.71	1.55–4.76	<0.001
Lack of follow-up of ED patients	0.21	0.12–0.37	<0.001
Overcrowding in ED	0.26	0.12–0.56	<0.001
Lack of medical staff	0.27	0.15–0.47	<0.001

ED: emergency department; HCW: healthcare workers; PCR: polymerase chain reaction; ^1^ Physician or nurse; ^2^ <30,000/year, 30,000–60,000/year, or >60,000.

## Data Availability

Materials include in appendixes are available from the corresponding author.

## Appendix A

This graphic was made from the public data source of Santé Publique France, Ministère de la santé (Ministry of Health).

Santé Publique France. COVID 19 point épidémiologique. Available online: https://www.santepubliquefrance.fr/recherche/#search=COVID%2019%20%20%20point%20epidemiologique&publications=données&regions=National&sort=date accessed on 9 January 2021.

## Appendix B. Emergency Department Vaccination Survey (English Version)

Please complete this questionnaire.

* Mandatory

1. E-mail address *
  Your email address
2. Please specify your department (01, 60, 75, 92,…) *
  Your answer
3. What is your status?
  Head of department/UF
  Hospital practitioner
  Other medical status
  Care/Health Executive
  IDE
4. Type of ED *
  Adult ED
  Pediatric ED
  Adult/Pediatric ED
5. Specify the type of facility *
  University hospital
  General Hospital
  Private non-profit
  Private for profit
6. Specify the number of annual visits *
  <30,000
  between 30,000 and 60,000
  more than 60,000
7. Would you say that your ED (multiple responses possible) *
  Is very often overcrowded
  Waiting times are long
  There is not enough downstream care in the hospital
  The premises are inadequate
  Patients are often in the emergency room waiting for a hospital bed
  None of the above
8. Your facility has an immunization center *.
  Select (Yes/No)
9. Your facility conducts annual flu vaccination campaigns among health care staff
  Select (Yes/No)
10. Your facility conducts annual influenza vaccination campaigns for the general population *
  Select (Yes/No)
11. Your facility has an infectious disease service *
  Select (Yes/No)
12. The ED conducts an annual flu vaccination campaign for its staff *
  Select (Yes/No)
13. The ED implements an influenza vaccination campaign for at-risk ED patients *
  Select (Yes/No)
14. The ED performs PCR testing at the virology lab for influenza diagnosis in the ED *
  Select (Yes/No)
15. The ED performs point-of-care PCR testing for influenza diagnosis in the ED *
  Select (Yes/No)
16. The ED performs point-of-care antigen testing for diagnosis of influenza in the ED
  Select (Yes/No)
17. The ED performs rapid tests for tetanus antibodies *
  Select (Yes/No)
18. The ED performs tetanus vaccination if rapid test is negative *
  Select (Yes/No)
19. It is the ED’ mission to vaccinate its staff against the flu * Select (Yes/No)
  Strongly disagree (0)–Strongly agree (10)
20. It is the ED’s mission to vaccinate its staff against Covid * Strongly disagree (0)
  Strongly disagree (0)–Strongly agree (10)
21. It is the ED’s mission to vaccinate ED patients with an indication for vaccination against influenza * Strongly disagree (0)
  Strongly disagree (0)–Strongly agree (10)
22. It is the role of the ED to vaccinate ED patients with an indication for vaccination against the flu * Strongly disagree (0)
  Strongly disagree (0)–Strongly agree (10)
23. In your opinion, what are the factors limiting influenza vaccination in the ED (please check only one answer per line: Yes/somewhat yes/somewhat no/no) *
  - Lack of patient follow-up
  - Overcrowding of activity in the ED
  - Lack of medical staff
  - Lack of nursing staff
  - Difficulty in obtaining the support of the health care team
  - Patient refusal
  - Lack of vaccine
  - Lack of patient follow-up
  - ED overcrowding
  - Lack of medical staff
  - Lack of nursing staff
  - Difficulty of adherence of the health care team
  - Patient refusal
  - Lack of vaccine
24. In your opinion, what are the factors limiting vaccination against Covid in the ED (please tick only one answer per line: Yes/rather yes/rather no/no) *
  - Lack of patient follow-up
  - Overcrowding of activity in the ED
  - Lack of medical staff
  - Lack of nursing staff
  - Difficulty in obtaining the support of the health care team
  - Patient refusal
  - Lack of vaccine
  - Lack of patient follow-up
  - ED overcrowding
  - Lack of medical staff
  - Lack of nursing staff
  - Difficulty of adherence of the health care team
  - Patient refusal
  - Lack of vaccine

**Enquête vaccination aux urgences (Version française)**

Merci de compléter de ce questionnaire.

* Obligatoire

1. Adresse e-mail *
  Votre adresse e-mail
2. Veuillez préciser votre département (01, 60, 75, 92, …) *
  Votre réponse
3. Quel est votre statut *
  Chef de service/UF
  Praticien Hospitalier
  Autre statut médical
  Cadre de Soins/Santé
  IDE
4. Type de SAU *
  SAU adultes
  SAU pédiatrique
  SAU adulte/pédiatrique
5. Préciser le type d’établissement *
  CHU
  CH
  Privé sans but lucratif
  Privé lucratif
6. Préciser le nombre de passages annuel *
  <30,000
  entre 30,000 et 60,000
  plus de 60,000
7. Vous diriez que votre service d’urgence (plusieurs réponses possibles) *
  Est très souvent surchargé
  Les délais d’attente sont longs
  L’aval des urgences dans l’hôpital est insuffisant
  Les locaux sont inadaptés
  Des patients sont souvent aux urgences en attente d’un lit hospitalier
  Aucune de ces réponses
8. Votre établissement dispose d’un centre de vaccination *
  Sélectionner (Oui/Non)
9. Votre établissement met en place des campagnes de vaccination antigrippale chaque année chez le personnel de santé *
  Sélectionner (Oui/Non)
10. Votre établissement met en place des campagnes de vaccination antigrippale chaque année chez la population générale *
  Sélectionner (Oui/Non)
11. Votre établissement a un service de maladies infectieuses *
  Sélectionner (Oui/Non)
12. Le SAU met en place une campagne de vaccination antigrippale de son personnel chaque année *
  Sélectionner (Oui/Non)
13. Le SAU met en place une campagne de vaccination antigrippale des patients à risque consultant aux urgences *
  Sélectionner (Oui/Non)
14. Le SAU réalise des tests PCR au laboratoire de virologie pour le diagnostic de la grippe aux urgences *
  Sélectionner (Oui/Non)
15. Le SAU réalise des tests PCR aux urgences (point of care) pour le diagnostic de la grippe aux urgences *
  Sélectionner (Oui/Non)
16. Le SAU réalise des tests antigéniques aux urgences (point of care) pour le diagnostic de la grippe aux urgences *
  Sélectionner (Oui/Non)
17. Le SAU réalise des tests rapides pour la recherche des anticorps anti-tétanos *
  Sélectionner (Oui/Non)
18. Le SAU réalise la vaccination anti-tétanique si le test rapide est négatif *
  Sélectionner (Oui/Non)
19. C’est la mission des urgences de vacciner contre la grippe son personnel *
  Pas du tout d’accord (0)–Tout à fait d’accord (10)
20. C’est la mission des urgences de vacciner contre la Covid son personnel *
  Pas du tout d’accord (0)–Tout à fait d’accord (10)
21. C’est la mission des urgences de vacciner contre la grippe les patients des urgences ayant une indication à la vaccination *
  Pas du tout d’accord (0)–Tout à fait d’accord (10)
22. C’est la mission des urgences de vacciner contre la Covid les patients des urgences ayant une indication à la vaccination *
  Pas du tout d’accord (0)–Tout à fait d’accord (10)
23. Pour vous, quels sont les facteurs limitant la vaccination anti-grippale aux urgences (attention, cochez une seule réponse par ligne: Oui/plutôt oui/plutôt non/non) *
  - Absence de suivi des patients
  - Surcharge d’activité aux urgences
  - Manque de personnel médical
  - Manque de personnel infirmier
  - Difficulté d’adhésion de l’équipe soignante
  - Refus des patients
  - Absence de vaccin
  - Absence de suivi des patients
  - Surcharge d’activité aux urgences
  - Manque de personnel médical
  - Manque de personnel infirmier
  - Difficulté d’adhésion de l’équipe soignante
  - Refus des patients
  - Absence de vaccin
24. Pour vous, quels sont les facteurs limitant la vaccination anti-Covid aux urgences (attention, cochez une seule réponse par ligne: Oui/plutôt oui/plutôt non/non) *
  - Absence de suivi des patients
  - Surcharge d’activité aux urgences
  - Manque de personnel médical
  - Manque de personnel Infirmier
  - Difficulté d’adhésion de l’équipe soignante
  - Refus des patients
  - Absence de vaccin
  - Absence de suivi des patients
  - Surcharge d’activité aux urgences
  - Manque de personnel médical
  - Manque de personnel Infirmier
  - Difficulté d’adhésion de l’équipe soignante
  - Refus des patients
  - Absence de vaccin
